# Effects of Cylindrospermopsin Producing Cyanobacterium and Its Crude Extracts on a Benthic Green Alga—Competition or Allelopathy?

**DOI:** 10.3390/md13116703

**Published:** 2015-10-30

**Authors:** Viktória B-Béres, Gábor Vasas, Dalma Dobronoki, Sándor Gonda, Sándor Alex Nagy, István Bácsi

**Affiliations:** 1 Environmental Laboratory, Department of Environment and Conservation, Hajdú-Bihar County Government Office, 16 Hatvan street, Debrecen H-4025, Hungary; E-Mail: beres.viktoria@gmail.com; 2 Department of Hydrobiology, University of Debrecen, Egyetem tér 1, Debrecen H-4032, Hungary; E-Mails: dalmadobronoki@gmail.com (D.D.); nagy.sandor.alex@science.unideb.hu (S.A.N.); 3 Department of Botany, University of Debrecen, Egyetem tér 1, Debrecen H-4032, Hungary; E-Mails: vasas.gabor@science.unideb.hu (G.V.); gondasandor@gmail.com (S.G.)

**Keywords:** cyanobacterium, eukaryotic alga, interaction, co-culturing, allelopathy, toxicity

## Abstract

Cylindrospermopsin (CYN) is a toxic secondary metabolite produced by filamentous cyanobacteria which could work as an allelopathic substance, although its ecological role in cyanobacterial-algal assemblages is mostly unclear. The competition between the CYN-producing cyanobacterium *Chrysosporum* (*Aphanizomenon*) *ovalisporum*, and the benthic green alga *Chlorococcum* sp. was investigated in mixed cultures, and the effects of CYN-containing cyanobacterial crude extract on *Chlorococcum* sp. were tested by treatments with crude extracts containing total cell debris, and with cell debris free crude extracts, modelling the collapse of a cyanobacterial water bloom. The growth inhibition of *Chlorococcum* sp. increased with the increasing ratio of the cyanobacterium in mixed cultures (inhibition ranged from 26% to 87% compared to control). Interestingly, inhibition of the cyanobacterium growth also occurred in mixed cultures, and it was more pronounced than it was expected. The inhibitory effects of cyanobacterial crude extracts on *Chlorococcum* cultures were concentration-dependent. The presence of *C*. *ovalisporum* in mixed cultures did not cause significant differences in nutrient content compared to *Chlorococcum* control culture, so the growth inhibition of the green alga could be linked to the presence of CYN and/or other bioactive compounds.

## 1. Introduction

Despite many studies addressing toxicological aspects of cyanobacterial metabolites, their physiological (ecological) role remains largely unclear, although some authors suggested that these metabolites may play a crucial role in allelopathy [1]. Allelopathy can be an important factor in the organization and formation of both planktonic and benthic algal assemblages in rivers and especially in lakes [1]. Allelopathic effects can appear not only in planktonic or in benthic assemblages, but also between planktonic and benthic taxa [2,3,4]. The interspecific relations between planktonic and benthic algal/cyanobacterial organisms are much more pronounced in shallow lakes with extended littoral regions, than in deep lakes or rivers [4]. Planktonic species can negatively affect the benthic assemblages both by their presence (e.g., by shading and nutrient uptake) and by allelopathic compounds. One common difficulty in studying allelopathy, particularly for field studies, is to separate it from competition [1]. Extrapolation of detailed data from co-culturing experiments could lead one step closer to the understanding of these interspecific relations.

Cyanobacteria are one of the most important producers of allelochemicals and toxins in freshwaters [5,6,7,8]. Toxicity of cyanobacterial compounds is well-proven, but there are many questions in connection of the allelopathic effects of these secondary metabolites. The concentration of cyanotoxins are usually low in the water bodies, except during cyanobacterial water blooms [9] and the ability of toxin production and excretion is species-, and even population/strain-dependent [1,10,11,12].

To date over a dozen species of filamentous cyanobacteria belonging to Nostocales and Oscillatoriales orders are known as potent producers of the alkaloid cylindrospermopsin (CYN) including, for example: *Cylindrospermopsis raciborskii* [13], *Umezakia natans* [14], *Chrysosporum* (*Aphanizomenon*) *ovalisporum* [15], *Chrysosporum* (*Aphanizomenon*) *flos-aquae* [16], *Chrysosporum* (*Aphanizomenon*) *gracile* [17,18], *Chrysosporum* (*Aphanizomenon*) *klebahnii* [19], *Raphidiopsis curvata* [20], *Raphidiopsis mediterranea* [21], *Chrysosporum* (*Anabaena*) *bergii* [22], *Dolichospermum planktonicum* (*Anabaena planctonica*) [23], *Anabaena lapponica* [24], *Lyngbya wollei* [25], and several strains of *Oscillatoria* sp. [26]. Most recently *Dolichospermum* (*Anabaena*) *mendotae* [27] and the soil cyanobacterium, *Hormoscilla pringsheimii* [28] were reported as CYN producers. Due to the wide geographical distribution of CYN producing cyanobacteria, the toxin occurs globally, it was reported from Australia and New Zealand, Asia, South and North America, and Europe [29]. CYN production and release by the producer species is strongly strain-dependent, intra- and extracellular CYN concentrations appear to depend on a multiple number of environmental factors, and seem to be pleiotropic. [30]. There is a lot of information about the accelerated spread of potentially CYN producing filamentous cyanobacteria both worldwide [29], and in Hungary, although CYN production of Hungarian isolates was not proved yet [31,32,33]. Despite this frequent appearance of these species (mainly *Cylindrospermopsis raciborskii* and *Chrysosporum ovalisporum*), only few data are available about the role and effects of CYN in algal assemblages [34,35,36,37,38]. Bar-Yosef *et al.* [35] suggested that CYN-producing *Chrysosporum* (*Aphanizomenon*) *ovalisporum* could affect the presence, growth, and alkaline phosphatase activity of eukaryotic algae in field. They reported that CYN-concentration increased significantly due to phosphate deficiency, but there was no exact information, whether extra- or intracellular toxin content changed positively. Others found that the effects of crude extract of CYN-producing cyanobacteria and purified CYN depended on the concentration and on the target species [34,36,38]. Low concentrations of cyanobacterial crude extract can stimulate the growth of eukaryotic algal species (*Chlorella vulgaris* [36], *Chlamydomonas reinhardtii*, *Chlorella vulgaris,* and *Nannochloropsis* sp. [38]), but higher concentrations of crude extract inhibited these algae significantly. Lower concentrations of CYN (1 and 5 μg·L^−1^) caused slight growth inhibition but significantly up-regulated alkaline phosphatase activity in a *Microcystis aeruginosa* strain [34].

This study focuses on the interspecific interactions of the filamentous CYN-producing cyanobacterium, *Chrysosporum ovalisporum* and the benthic green alga, *Chlorococcum* sp. The interspecific relations (including allelopathy) between planktonic and benthic algal/cyanobacterial organisms could be pronounced in shallow lakes with extended littoral region, particularly during a cyanobacterial bloom. Till now, no benthic taxa were employed in studies investigating the effects of CYN on eukaryotic algae [34,35,36,37,38]. The genus *Chlorococcum* is cosmopolitan. Though primarily it is an edaphic alga, it has been reported from such diverse habitats as hot springs in Central Asia and soils collected in Antarctica. Freshwater, marine, and aerial isolates have been recorded [39]. Although according to our knowledge there are no literature data about the co-occurring of *C. ovalisporum* and *Chlorococcum* sp.; there are informal reports about the coexistence of these genera from several Hungarian standing waters. The growth and nutrient uptake were investigated in mixed cultures, and the effects of cyanobacterial crude extract on *Chlorococcum* sp. were tested in two ways: The green alga was treated with cell debris-containing (CDC) crude extracts of the cyanobacterium (CDC crude extract-treated cultures). The effects of the centrifuged, so called “cell debris-free” (CDF) crude extract of *C. ovalisporum* to *Chlorococcum* sp. were also studied (CDF crude extract-treated cultures).

Our hypotheses were the following: (i) during co-culturing, growth inhibition of the green alga increase with the increasing ratio of cyanobacteria, because of competition for nutrients and possible allelopathic effect of cyanobacterial compounds (mainly CYN); (ii) it is known from the literature, that low cyanotoxin concentrations could have stimulatory effects, so we assumed, that the lowest concentration of both kinds of crude extracts stimulate the green algal populations, only higher content of crude extracts (if any) cause growth inhibition; (iii) inhibitor effects of the cell debris containing (CDC) crude extract is higher than that of the cell debris-free (CDF) crude extract, because of the shading effect of the cell debris and because of possible presence of inhibitor compounds other than CYN (fatty acids, polysaccharides, or enzymes).

## 2. Results

### 2.1. Coexistence of Chrysosporum ovalisporum and Chlorococcum sp. in Mixed Cultures

#### 2.1.1. Changes of Eukaryotic Algal and Cyanobacterial Cell Numbers

**Figure 1:**
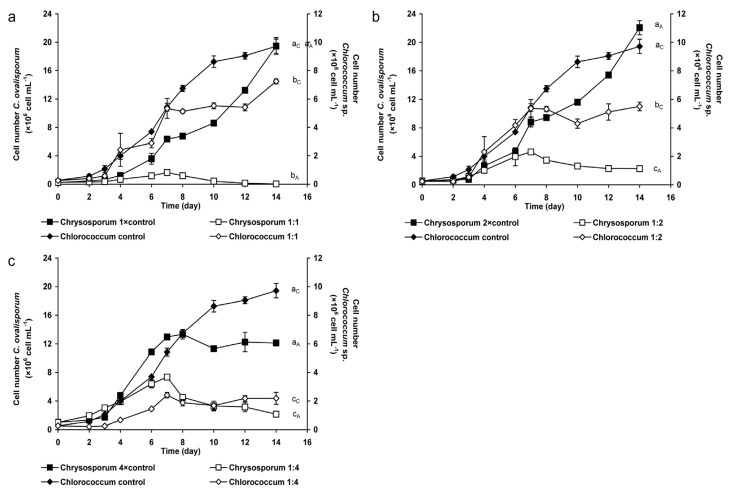
Changes of cell numbers in control (containing *Chlorococcum* sp. or *C. ovalisporum* cells only) and in mixed (containing *Chlorococcum* sp. and *C. ovalisporum* cells, as well) cultures. Initial *Chlorococcum* sp. cell numbers were 0.2 × 10^6^ mL^−1^ in control and mixed cultures in all cases (**a**–**c**). Initial *C. ovalisporum* cell numbers were in control and mixed cultures: (**a**) 0.2 × 10^6^ mL^−1^ (1:1 ratio); (**b**) 0.4 × 10^6^ mL^−1^ (1:2 ratio); and (**c**) 0.8 × 10^6^ mL^−1^ (1:4 ratio). Significant differences among the growth of *Chlorococcum* sp. cultures are indicated with a_C_–c_C_; significant differences among the growth of *C. ovalisporum* cultures are indicated with a_A_–c_A_.

There were significant differences between the growth of the green alga in control and in mixed cultures (*p* < 0.05; Figure 1a). Furthermore, growth of *Chlorococcum* differed significantly from each other in mixed cultures 1:1 and 1:4, as well as 1:2 and 1:4 (*p* < 0.05). The growth of the green alga was continuous but with a lower extent compared to the control culture on the second week in mixed cultures with 1:1 and 1:2 ratio. The cell number of *Chlorococcum* was 25% and 44% lower, respectively, than the cell number in control culture on the last (14th) day (Figure 1a,b). The decrease of the green algal cell number was the most conspicuous in the mixed culture with 1:4 ratio, growth of *Chlorococcum* sp. was inhibited during the whole experiment (Figure 1c). The cell number of the benthic green alga was significantly lower (*p* < 0.05) in this mixed culture than both in control culture and in the other mixed cultures (Figure 1c).

The growth of *C. ovalisporum* was significantly lower in mixed cultures than in control ones (*p* < 0.05). Significant differences were observed in mixed cultures between 1:1 and 1:2, and also between 1:1 and 1:4 ratios (*p* < 0.05). The cell number of the cyanobacterium increased in mixed cultures with 1:1 and 1:2 ratios in the first week (Figure 1a,b). After that, the growth of *C. ovalisporum* decreased to the end of the experiment in both cultures, cell number of the cyanobacterium was 99% and 90% lower in these mixed culture than in control culture on the 14th day (Figure 1a,b). Similar phenomenon was observed in mixed culture with 1:4 ratio, cyanobacterial cell number was 82% lower in this mixed culture than cell number in the control culture at the end of the experiment (Figure 1c).

#### 2.1.2. Changes of Nitrate- and Phosphate Content of the Medium

**Figure 2:**
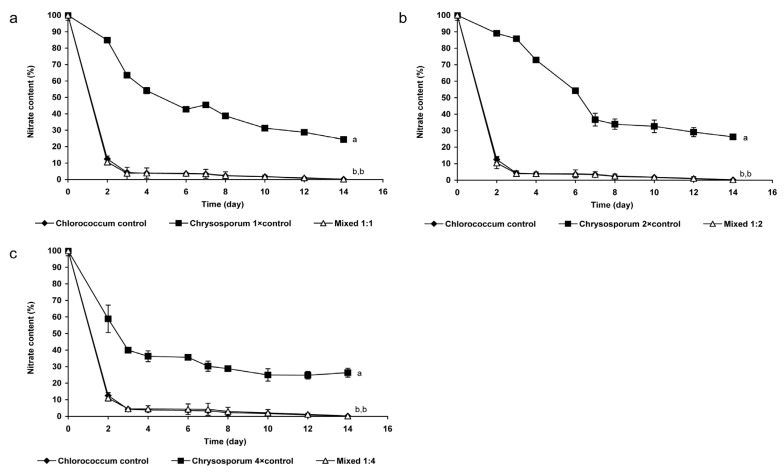
Changes of nitrate-concentration in mixed and control cultures (**a**) 0.2 × 10^6^ cell·mL^−1^ *Chlorococcum* sp. and 0.2 × 10^6^ cell·mL^−1^ *C. ovalisporum*; (**b**) 0.2 × 10^6^ cell·mL^−1^ *Chlorococcum* sp. and 0.4 × 10^6^ cell·mL^−1^ *C. ovalisporum*; and (**c**) 0.2 × 10^6^ cell·mL^−1^ *Chlorococcum* sp. and 0.8 × 10^6^ cell·mL^−1^ *C. ovalisporum*. Significant differences are indicated with different lowercase letters.

Nitrate-uptake in monoalgal *Chrysosporum* cultures was slower than those in mixed cultures or in *Chlorococcum* controls. The changes of nitrate-content of mixed cultures differed significantly from that of *C. ovalisporum* control cultures in all cases (*p* < 0.05; Figure 2). In contrast, the changes of nitrate-content of mixed cultures did not differ significantly from that of the *Chlorococcum* control culture, almost 90% of nitrate was taken up to the second day (Figure 2).

**Figure 3:**
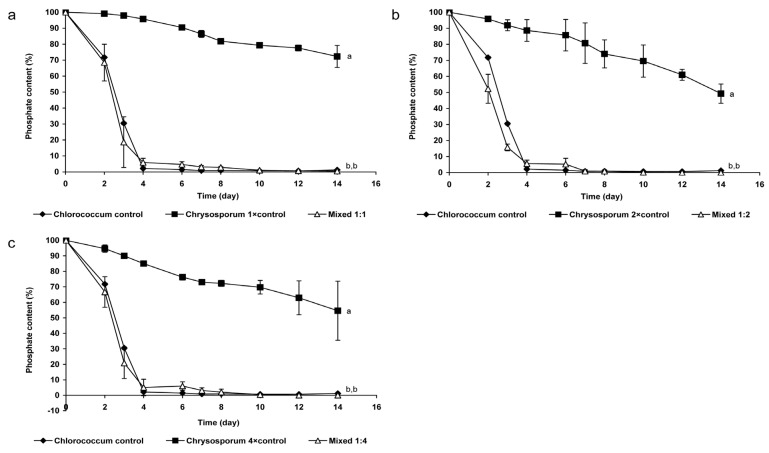
Changes of phosphate-concentration in mixed and control cultures (**a**) 0.2 × 10^6^ cell·mL^−1^ *Chlorococcum* sp. and 0.2 × 10^6^ cell·mL^−1^ *C. ovalisporum*; (**b**) 0.2 × 10^6^ cell·mL^−1^ *Chlorococcum* sp and 0.4 × 10^6^ cell·mL^−1^ *C. ovalisporum*; (**c**) 0.2 × 10^6^ cell·mL^−1^ *Chlorococcum* sp and 0.8 × 10^6^ cell·mL^−1^ *C. ovalisporum*. Significant differences are indicated with different lowercase letters.

Generally, phosphate-content decreased slower, *i.e.*, phosphate-uptake was significantly lower (*p* < 0.05) in control cultures of *C. ovalisporum* than in the cases of the different mixed cultures (Figure 3). The cyanobacterium did not take up more than 50% of available phosphate in control cultures (Figure 3). In contrast, phosphate-uptake reached 90%–100% in the cases of green algal control culture and also in mixed cultures. Decrease of phosphate-content of green algal control culture and mixed cultures were not significantly different (Figure 3).

#### 2.1.3. Changes of Extra- and Intracellular CYN Content

**Figure 4:**
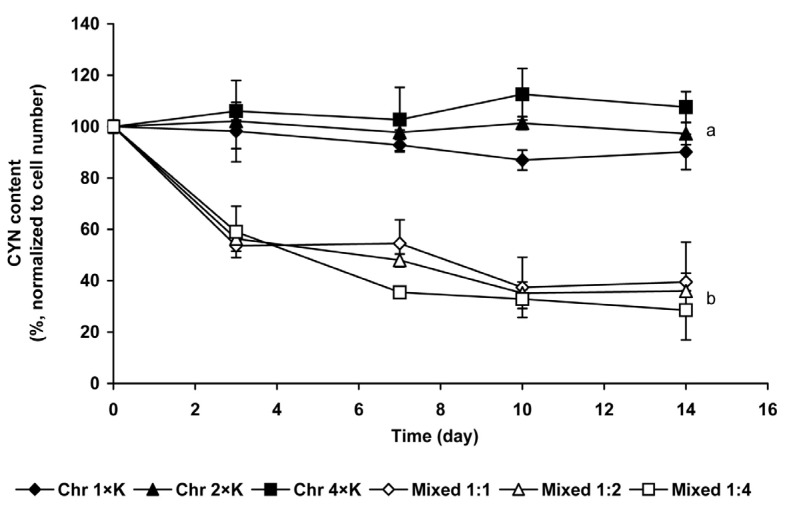
Changes of intracellular CYN-content (%) in mixed and in *C. ovalisporum* control cultures. The obtained data were normalized to the zero time controls which were chosen to 100% on basis of cell number. Significant differences among the CYN content changes of control and mixed cultures are indicated with different lowercase letters.

Extracellular CYN concentration was under the limit of detection in *Chrysosporum* control cultures and in mixed cultures, *i.e.*, taking into consideration the concentrating step of the method (see in Experimental Section), the culturing medium contained maximum 0.1 µg·mL^−1^ cyanotoxin. Based on capillary electrophoretic measurements, the CYN content was 2.15 × 10^−4^ ng per cell. Intracellular CYN content significantly (*p* < 0.05) decreased in mixed cultures compared to *C. ovalisporum* controls; it was 60%–70% lower on the 14th day than at the beginning of the experiment. Intracellular CYN content of mixed cultures did not differ significantly from each other (Figure 4).

### 2.2. Chlorococcum sp. Cultures Treated with Cyanobacterial Crude Extract

#### 2.2.1. Changes of *Chlorococcum* sp. Cell Number

**Figure 5:**
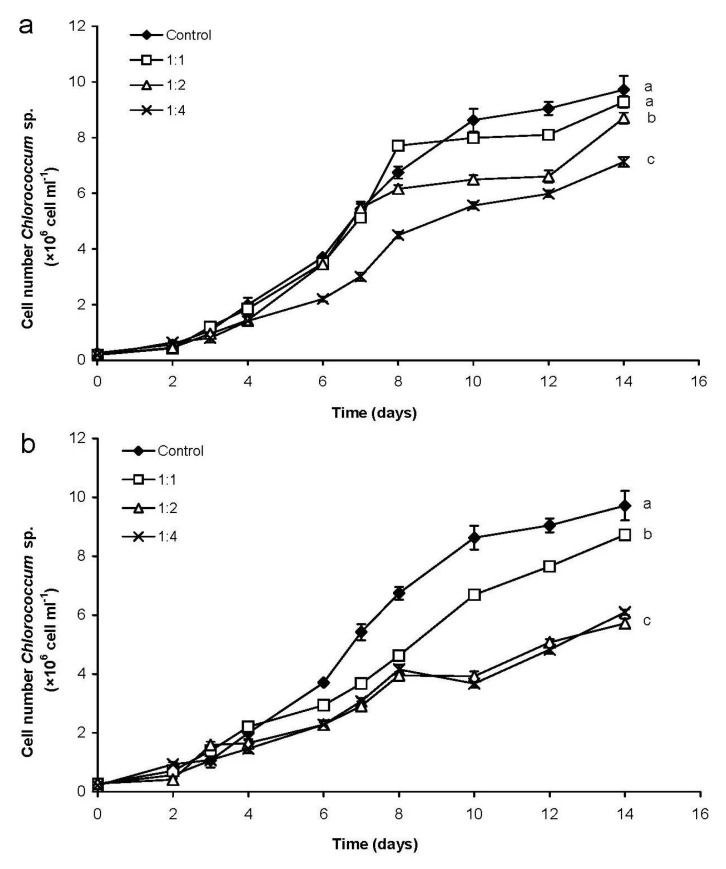
Changes of cell number in *C. ovalisporum* crude extract treated *Chlorococcum* cultures. (**a**) Cultures treated with CDC crude extract; and (**b**) cultures treated with CDF crude extract. Significant differences are indicated with different lowercase letters.

The increase of cell number was significantly lower in the CDC crude extract treated cultures only in the case of 1:4 ratio (*p* < 0.05; Figure 5a). Changes of cell number of the green algal cultures treated with different amount of CDC crude extract differed from each other significantly, except of 1:1 and 1:2 cultures (*p* < 0.05; Figure 5a). The inhibition of growth was clear in culture treated with CDC crude extract in 1:2 ratio from the seventh day, cell number of this CDC crude extract treated culture was 10.5% lower than control culture on the last day of the experiment (Figure 5a). The most conspicuous inhibition was detected in culture treated with CDC crude extract in 1:4 ratio, cell number of this treated culture was 27% lower than in control culture on the 14th day (Figure 5a).

Growths of the green alga were significantly lower in cultures treated with different concentrations of CDF crude extract than in control culture (*p* < 0.05; Figure 5b). The cell number changes of CDF crude extract-treated cultures differed significantly from each other (*p* < 0.05; Figure 5b), except 1:2 *vs.* 1:4 ratios. The cell number of cultures treated with CDF crude extract in 1:1 ratio was 10% lower on the 14th day than in the case of control culture (Figure 5b). Clear growth inhibition was detected in the cases of green algal cultures treated with CDF crude extract in 1:2 and 1:4 ratio from the fourth day. Cell numbers of these cultures were significantly lower (*p* < 0.05) on the last day (37% and 41%, respectively) than in control cultures (Figure 5b).

#### 2.2.2. Changes of Nitrate- and Phosphate-Content of the Medium

**Figure 6:**
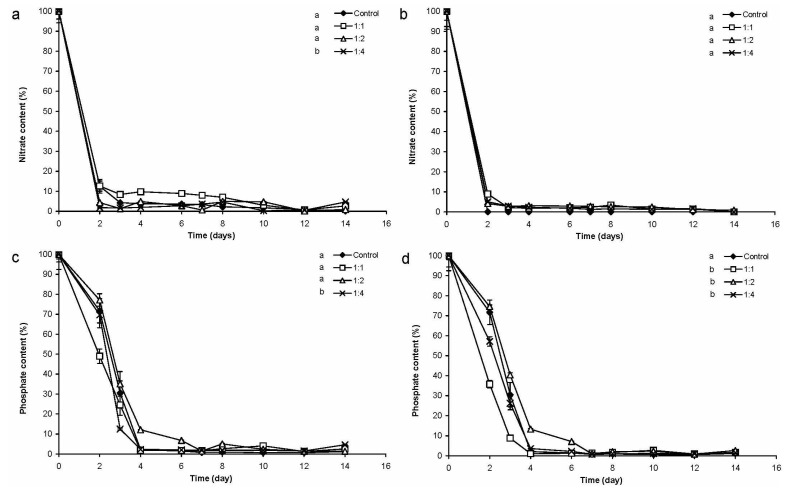
Changes of nitrate-concentration and phosphate-concentration in *C. ovalisporum* crude extract treated *Chlorococcum* cultures. (**a**) Nitrate-concentration changes in cultures treated with CDC crude extract; (**b**) nitrate-concentration changes in cultures treated with CDF crude extract; (**c**) phosphate-concentration changes in cultures treated with CDC crude extract; and (**d**) phosphate-concentration changes in cultures treated with CDF crude extract. Significant differences are indicated with different lowercase letters.

The nitrate-content decreased drastically to the second day both in control and in CDC crude extract treated cultures, less than 20% of the initial nitrate-content remained in the culturing media in every cases. There were no significant differences among these decreases (Figure 6a). Similarly, there were strong decreases of nitrate-content in CDF crude extract treated cultures to the second day. There were no significant differences between the changes of nitrate-content in all cultures (Figure 6b).

Phosphate-content of the cultures decreased slower than nitrate content, but only 2%–10% of the initial phosphate concentration remained to the fourth day in all cultures. Changes of phosphate content did not differ significantly from each other in all cultures in CDC crude extract treated (Figure 6c) and in CDF crude extract-treated cultures (Figure 6d).

#### 2.2.3. Changes of CYN Concentration

Based on the intracellular CYN content of *C. ovalisporum*, the initial CYN contents were calculated as 0.04, 0.09, and 0.17 µg·mL^−1^ for 1:1, 1:2, and 1:4 treatments, respectively. The toxin concentration was possible to verify only in the case of 1:4 crude extract treated culture by the applied method (see in Experimental Section). The toxin content of the culturing medium was under the limit of detection (0.1 µg·mL^−1^ applying tenfold concentration) after the first day both in CDC crude extract and CDF crude extract-treated cultures, therefore further changes were not traceable.

### 2.3. Comparison the Changes of Different Chlorococcum Cultures

The *Chlorococcum* cell numbers of mixed cultures were significantly lower (*p* < 0.05) than in CDC crude extract-treated cultures, except in the case of 1:2 ratio. The *Chlorococcum* cell numbers of mixed and CDF crude extract-treated cultures showed significant difference only in the case of 1:4 ratio. Cultures treated with CDF crude extracts contained significantly lower numbers of *Chlorococcum* cells than CDC crude extract-treated cultures only in the case of 1:2 ratio (*p* < 0.05). Growth inhibition increased with the increasing amount of living cyanobacteria or cyanobacterial extracts. Living cyanobacteria caused the strongest inhibition, while growth inhibition in culture treated with CDF crude extracts was stronger, than in culture treated with CDC extracts (see above where significant differences occurred).

Changes in nitrate content of the mixed cultures, CDC crude extract treated cultures and CDF crude extract-treated culture did not show significant differences from each other in all cases. Similar phenomenon was observed in the case of phosphate content changes.

## 3. Discussion

### 3.1. Coexistence of Chrysosporum ovalisporum and Chlorococcum sp. in Mixed Cultures

In spite of the fact that CYN can affect negatively algal growth and survival [36,37,38], the effects of CYN-containing cyanobacterial water blooms to algal assemblages are still unclear. According to our knowledge, only one field experiment is available on the effects of CYN-producing filamentous cyanobacteria to eukaryotic algal taxa [35]. In the light of current literature, we hypothesized that the presence of cyanobacterium inhibits green algal growth both due to competition and the presence of the extracellular CYN. Our results confirmed this suggestion: growth of *Chlorococcum* sp. was significantly lower in mixed cultures than in control cultures, decrease of green algal cell number was much more intense in the presence of higher initial cell number of the cyanobacterium. Furthermore, growth changes of *Chlorococcum* sp. were strongly connected to the growth of the cyanobacterium in the given mixed cultures. These results could suggest both competition and allelopathy. For a correct explanation, the changes in nutrient uptake and extracellular CYN content should be taken into account both in mixed and in control cultures.

The results showed more intensive nitrate-uptake in *Chlorococcum* sp. control cultures than in *C. ovalisporum* control cultures. *Chlorococcum* seemed to determine the speed of nitrate-uptake in mixed cultures. Similar phenomena were observed also in the case of phosphate-uptake. On the basis of these facts, mixed cultures and *Chlorococcum* control culture can be considered as nitrogen and phosphorous limited cultures already from the fourth day. This could be one reason of growth inhibition of the green alga in mixed cultures, but it has to be taken into consideration that the growth of green algal control culture was not inhibited. The presence of *C. ovalisporum* in mixed cultures did not cause significant differences in nutrient content compared to *Chlorococcum* control culture, so the growth inhibition of the green alga could be linked to some other reasons.

The effects of abiotic factors on the production of CYN are quite well-known both from field studies [40,41,42], and from laboratory experiments (temperature [43]; phosphate-, sulphate-, and nitrate starvation [44,45]; and temperature and light intensity [46,47]. It seems, that extracellular CYN-content could change in a wide range due to different abiotic factors (*Cylindrospermopsis raciborskii*: 39 × 10^−3^ μg·mL^−1^ [48]; *Chrysosporum* (*Aphanizomenon*) *gracile* and *C*. (*A.*) *flos-aquae*: 0–11.8 × 10^−3^ μg·mL^−1^ [17]; and *C*. (*A.) ovalisporum*: 4–120 × 10^−3^ μg·mL^−1^ [49]; 23.3 × 10^−3^ μg·mL^−1^ [42]). In contrast, there is no available information about the changes of extracellular CYN-concentration due to biotic factors. Bar-Yosef *et al.* [35] reported increase of CYN in the presence of high eukaryotic algal density in field, but no exact information was given about the location of the increasing toxin amount (extra- or intracellular CYN). In our experiments, extracellular CYN level was below 0.1 μg·mL^−1^ in *Chrysosporum* control cultures and in mixed cultures. Furthermore, intracellular CYN decreased in mixed cultures. These decreases are in accordance with our previous findings about CYN content changes of the used *Chrysosporum* strain under nutrient limitation [44,45]. The other potential reason of measuring low amount of extracellular CYN could be that due to the rich matrix particulate (but otherwise effective) CYN [17] occurred, which was not detectable by our capillary electrophoretic method developed for free dissolved CYN measurements. Based on the showed results (nutrient content changes, intra- and extracellular CYN content changes) we suggest that the decrease of the cell number of the green alga in mixed cultures occurred also due to the presence of other bioactive compounds than only CYN. However, numerous studies show that pure CYN are less toxic than extracts of CYN producers e.g., in the case of aquatic plants [50] or eukaryotic alga [35]. Therefore, effects of both dissolved and particulated CYN have to be taken into consideration, especially in view of the sensitivity of *Chlorococcum* to extracts with low dissolved CYN concentrations (see below).

It was not surprising that the cell number of *C. ovalisporum* was lower in mixed cultures than in control cultures, because the fact that eukaryotic algae are able to negatively affect cyanobacterial growth is known [51,52]. However, the differences of cyanobacterial growth in mixed and control cultures were much higher than they were expected. The interspecific competition for nutrients could be one explanation of this phenomenon. Both nitrate- and phosphate-uptake of mixed cultures were more similar to the nutrient uptake of *Chlorococcum* sp. control cultures than that of the different *Chrysosporum* control cultures, and the forming nutrient-poor circumstances definitely affected the growth of the cyanobacterium. Another possible explanation could be that *Chlorococcum* species are able to produce a range of metabolites with antibacterial, antifungal, and algaecide effects [2,53,54,55]. Although direct allelopathic effects of these algae have not been detected yet, it is possible that our *Chlorococcum* strain produced some metabolites with anticyanobacterial effects, which contributed to the observed cell number decreases of the cyanobacterium. This presumption needs further investigations.

### 3.2. Chlorococcum sp. Cultures Treated with Cyanobacterial Crude Extract

Crude extracts imitate well field conditions [51] when a cyanobacterial water bloom collapses. It is pertinent to emphasize that there are huge differences between the types of the used extracts (CDC crude extracts containing microcystin variants [51,56]; CDF crude extracts of microcystins and cylindrospermopsin [38]; unknown type of CYN-containing crude extracts [36]). In our experiments the effects of different types of crude extracts were compared.

As it is already mentioned, only a few data are available about the effects of CYN-containing cyanobacterial crude extracts to eukaryotic algal growth [36,37,38,39]. These studies presented concentration-dependent inhibitory or stimulatory effects of cyanobacterial crude extract. We hypothesized that the lowest concentrations of both CDC and CDF crude extracts stimulate the growth of the green alga, while the highest concentrations of crude extracts may inhibit the increase of *Chlorococcum* sp. cultures. Our presumptions were partially confirmed by the results. The growth of *Chlorococcum* sp. was significantly lower in all cases than in control cultures, independently of the type of crude extracts. The inhibitory effects depended on the concentration of the crude extracts. Similarly to the results of Campos *et al.* [36] and Pinheiro *et al.* [38], we also found, that higher crude extract concentrations caused a higher decrease in cell number than lower concentrations of cyanobacterial extracts. On the other hand, stimulatory effects were not observed even in the case of the lowest crude extract concentration, independently of the type of crude extracts.

Our further presumption was that the inhibitory effects of CDC crude extracts will be more pronounced than in cases of CDF crude extracts, because of the shading effect of the cell debris and because of the possible presence of inhibitor compounds other than CYN (fatty acids, polysaccharides or enzymes). The results did not confirm this presumption; in fact, just the opposite was found. Despite the fact that the toxin content was the same in the different types of crude extracts, the inhibitory effects were much more pronounced in the cases of CDF crude extract treated green algal cultures than in CDC crude extract treated ones. Water soluble bioactive compounds other than CYN could be present in both types of crude extracts. It is possible that the effects of water-soluble CYN and other water soluble bioactive compounds were expressed better in the CDF crude extracts than in CDC crude extracts. Cell debris might serve as a surface temporarily binding part of dissolved bioactive compounds, leading to decreased inhibitory effects. Furthermore, the cell debris could contain higher amount of different beneficial compounds associated to the particles (e.g., nutrients and hormone-like compounds [57,58,59,60]) than CDF crude extracts. These compounds could help to survive or to grow of green algal population in CDC extract-treated cultures. Of course, further studies are needed to confirm this hypothesis.

The effects of cyanobacterial crude extracts primarily depend on the toxin concentration [36,38]. We found that even the lowest concentration of crude extract (calculated as 0.043 μg·mL^−1^ initial CYN-content on the basis of intracellular toxin content) inhibited the growth of the green alga. Similarly, Campos *et al.* [36] found that the cell number of *Chlorella vulgaris* cultures decreased in the presence of crude extract containing 0.032 μg·mL^−1^ CYN. In contrast, Pinheiro *et al.* [38] reported inhibitory effects of crude extracts on *Chlorella vulgaris*, *Chlamydomonas reinhardtii*, and *Nannochloropsis* sp. only in the cases of the highest CDF crude extract concentration (2.5 μg·mL^−1^ CYN), but the ratio of the inhibition was different on the three taxa. The marine *Nannochloropsis* sp. was the most sensitive to the CYN-containing cyanobacterial extracts. The habitats of CYN-producing cyanobacteria and *Nannochloropsis* green alga are quite different, so the authors suggested that effective defense mechanisms probably could not develop in *Nannochloropsis* sp. [38]. Based on this suggestion, we supposed that the sensitivity of *Chlorococcum* sp. to CYN-containing crude extracts was due to similar reasons: although potentially CYN-producing filamentous cyanobacteria were found in Hungarian lakes and ponds, CYN has not been detected in Hungary yet [32]. Our *Chlorococcum* sp. was isolated from a Hungarian little pond, this could be one possible reason, why this green alga strain is sensitive to CYN-containing crude extracts.

## 4. Experimental Section

### 4.1. Strains and Culturing Conditions

The cylindrospermopsin (CYN) producer *Chrysosporum ovalisporum* strain (ACCDH-UD1001) is a derivative of the strain ILC-164 isolated from Lake Kinneret, Israel. The *Chlorococcum* sp. (ACCDH-UD1002) was isolated from a shallow pond in Eastern Hungary. The strains are maintained in the Algal Culture Collection, Department of Hydrobiology at the University of Debrecen.

The experiments were carried out in Erlenmeyer flasks (with a final volume of 400 mL) in Jaworski’s medium [61], bubbled with sterile air under continuous irradiation (80 µmol photons m^−2^·s^−1^) at 24 °C. The duration of investigations was 14 days.

#### 4.1.1. Mixed Cultures

One-week-old cultures (in the exponential growth phase) served as inoculums, the inoculating cell number of *Chlorococcum* sp. was 0.2 × 10^6^ cell·mL^−1^ in all cultures. Inoculating cell numbers of *C. ovalisporum* were 0.2 × 10^6^ cell·mL^−1^, 0.4 × 10^6^ cell·mL^−1^ or 0.8 × 10^6^ cell·mL^−1^, referred to as 1:1, 1:2 and 1:4 ratios throughout the paper, respectively. The final volume of the cultures was 400 mL. Samples of 1 mL were taken for cell number counting and nutrient (nitrate and phosphate) measurements. Cell numbers were counted in a Bürker chamber on 400× magnification using an Olympus BX50F-3 fluorescent microscope (Olympus Optical Co., Ltd, Tokyo, Japan).

#### 4.1.2. Crude Extract Treated Cultures, Preparation of Cyanobacterial Crude Extract

The inoculating cell number of *Chlorococcum* sp. was 0.2 × 10^6^ cell·mL^−1^ in treated cultures and in the *Chlorococcum* control culture. Crude extracts with cell debris (CDC crude extracts) were added in amounts containing the appropriate cell number of *C. ovalisporum* to reach 1:1, 1:2, and 1:4 ratios. Cell debris-free crude extracts (CDF crude extracts) were centrifuged (16,200× *g*, 5 min.; 24 °C, Heraeus Fresco 17 centrifuge, Thermo Fisher Scientific Inc., Waltham, MA, USA), supernatant was added to the *Chlorococcum* cultures in the same amount as in the case of CDC crude extracts to reach 1:1, 1:2, and 1:4 ratios.

For the preparation of crude cyanobacterial extract, 14-day-old culture of *C. ovalisporum* was centrifuged (6000× *g*, 10 min, 4 °C, Beckman Avanti J-25 centrifuge, Beckman Coulter Inc., Pasadena, CA, USA), the cell pellet was lyophilized (Christ Alpha 1–2 LD plus, Martin Christ Gefriertrocknungsanlagen GmbH, Osterode am Harz, Germany) and stored at −20 °C until further experiments. The lyophilized cyanobacterial cell mass was resuspended in sterile distilled water; the cells were disrupted by freezing and thawing at least four times (freezing was achieved keeping the cyanobacterial cell mass in liquid nitrogen until its total solidification, thawing was carried out at room temperature, until total liquefaction of the sample). The suspension was used without centrifugation in the case of CDC crude extract, and was centrifuged as described above in the case of CDF crude extract.

### 4.2. Nutrient Measurements

After cell number counting, samples were centrifuged (16,200× *g*, 5 min.; 24 °C, Heraeus Fresco 17 centrifuge, Thermo Fisher Scientific Inc., Waltham, MA, USA), the supernatants were removed and stored at −20 °C before further processing. Measurement of nitrate concentration was implemented from 400 µL aliquot of the supernatant, using the salicylic acid colorimetric method [62]. Inorganic dissolved phosphorus concentrations were measured from 100 µL aliquot of the supernatant by the acidic molybdate method [63]. Spectrophotometric measurements were done in a Hach Lange DR 6000 UVVIS spectrophotometer (Hach Company, Loveland, CO, USA). The nutrient contents of the culturing media were given in percentage, 100% were the amounts measured at the beginning of the experiments.

### 4.3. Quantification of Cylindrospermopsin Content of C. ovalisporum Cells and Culturing Medium

Three milliliters of culture samples were centrifuged (16,200× *g*, 5 min.; 24 °C, Heraeus Fresco 17 centrifuge, Thermo Fisher Scientific Inc., Waltham, MA, USA) and the pellets and supernatants were lyophilized separately. The CYN content was determined by capillary electrophoresis (PrinCE-C 700, Prince Technologies, Emmen, The Netherlands, fused silica capillary with 80 cm total length and 50 µm i.d.; 100 mbar 0.15 min hydrodynamic injection, +25 kV voltage, 20 min running time). For the plotting of CYN content of the cells, the obtained data were normalized to the zero time controls which were chosen to 100% on basis of cell number. Lyophilized supernatants were re-suspended in 300 µL 10% propanol. After sonication and centrifugation, CYN measurement was conducted as in the case of the cells. Six-point calibration curves (10–160 µg·mL^−1^) of CYN dissolved in 10% propanol were used to determine the linear range of the measurement. CYN standard was purified in our laboratory according to Vasas *et al.*, 2002 [64]. Limit of detection (LOD) was 1 µg·mL^−1^, limit of quantification (LOQ) was 2.5 µg·mL^−1^ for the applied method. Maximum ten-fold concentrations were applicable in the case of the supernatants, so 0.1 and 0.25 µg·mL^−1^ were the minimum amount of CYN for detection and quantification, respectively.

### 4.4. Statistical Analysis

All experiments were done in triplicate. One-way ANCOVA was used to determine the significances among the tendency-differences of curves of control and treated cultures [65,66]. Data are reported by mean values ± standard deviations.

## 5. Conclusions

Our study introduces direct interaction-experiments of CYN-producing *Chrysosporum ovalisporum* and eukaryotic alga in mixed cultures. In addition, the effects of cell debris-containing and cell debris-free crude extracts on the growth and nutrient-uptake of green algal cultures were compared.

The results of the mixed cultures suggested that the presence of bioactive compounds beside CYN were the main factors influencing the coexistence of the studied cyanobacterial and green algal populations. However, the decrease of cyanobacterial cell number was much higher in mixed cultures than was expected. This phenomenon implied the potential presence of anticyanobacterial compounds produced by *Chlorococcum* sp., with the additional competition for nutrients. Detection and specification of these hypothetic compounds require further studies.

Surprisingly, the inhibitory effects of cell debris-free crude extracts were more pronounced than that of cell debris-containing extracts. Cell debris may serve as a surface, decreasing the amount of dissolved CYN and other bioactive compounds; or cell debris could contain facilitating compounds, which could help the growth of the green alga even in the presence of inhibitor molecules. Proof of these presumptions needs further investigations.

The studied *Chlorococcum* sp. seemed to be sensitive even to relatively low CYN concentrations, which suggests that CYN, if released, could have a role in the formation even of benthic assemblages, especially in shallow ponds with large littoral regions.
